# RBC deformability and amino acid concentrations after hypo-osmotic challenge may reflect chronic cell hydration status in healthy young men

**DOI:** 10.1002/phy2.117

**Published:** 2013-10-23

**Authors:** Jodi D Stookey, Alexis Klein, Janice Hamer, Christine Chi, Annie Higa, Vivian Ng, Allen Arieff, Frans A Kuypers, Sandra Larkin, Erica Perrier, Florian Lang

**Affiliations:** 1 Children's Hospital Oakland Research InstituteOakland, California; 2 Danone ResearchPalaiseau, France; 3 Pediatric Clinical Research Center, Children's Hospital & Research Center OaklandOakland, California; 4 Department of Medicine, University of California San FranciscoSan Francisco, California; 5 University of TuebingenTuebingen, Germany

**Keywords:** Amino acid, arginine, biomarker, cell hydration, glutamate, healthy adults, histidine, RBC deformability, water intake

## Abstract

Biomarkers of chronic cell hydration status are needed to determine whether chronic hyperosmotic stress increases chronic disease risk in population-representative samples. In vitro, cells adapt to chronic hyperosmotic stress by upregulating protein breakdown to counter the osmotic gradient with higher intracellular amino acid concentrations. If cells are subsequently exposed to hypo-osmotic conditions, the adaptation results in excess cell swelling and/or efflux of free amino acids. This study explored whether increased red blood cell (RBC) swelling and/or plasma or urine amino acid concentrations after hypo-osmotic challenge might be informative about relative chronic hyperosmotic stress in free-living men. Five healthy men (20–25 years) with baseline total water intake below 2 L/day participated in an 8-week clinical study: four 2-week periods in a U-shaped A-B-C-A design. Intake of drinking water was increased by +0.8 ± 0.3 L/day in period 2, and +1.5 ± 0.3 L/day in period 3, and returned to baseline intake (0.4 ± 0.2 L/day) in period 4. Each week, fasting blood and urine were collected after a 750 mL bolus of drinking water, following overnight water restriction. The periods of higher water intake were associated with significant decreases in RBC deformability (index of cell swelling), plasma histidine, urine arginine, and urine glutamic acid. After 4 weeks of higher water intake, four out of five participants had ½ maximal RBC deformability below 400 mmol/kg; plasma histidine below 100 μmol/L; and/or undetectable urine arginine and urine glutamic acid concentrations. Work is warranted to pursue RBC deformability and amino acid concentrations after hypo-osmotic challenge as possible biomarkers of chronic cell hydration.

## Introduction

Insufficient water intake causes hyperosmotic stress on cells, cell shrinkage, and compensatory responses to restore cell volume. The compensatory responses, including urine concentration (Star 1990), insulin resistance (Bratusch-Marrain and DeFronzo 1983; Berneis et al. 1999), and increased inflammatory response (McKenzie et al. 1999; Judelson et al. 2008), are risk factors for prevalent chronic diseases affecting Western societies, including kidney and cardiovascular disease (Perucca et al. 2007; Torres et al. 2011), diabetes and Alzheimer′s disease (Lue et al. 2012). Low water intake is associated with increased chronic disease risk (Manz 2007). It is unknown if or how chronic hyperosmotic stress on cells mediates effects of low water intake, or if there is a role for chronic cell hydration in disease prevention or treatment.

To pursue relationships between chronic hyperosmotic stress on cells and chronic disease, measures of *chronic* cell hydration status are needed. There is particular need for measures that are feasible for use in population-representative samples to support public health inference about chronic disease risk. Available indices of *acute* cell hydration state, such as elevated serum or urine osmolality at a point in time, may misrepresent usual status as they reflect a narrow window of time (e.g., 2–4 h) before the sampling (Nose et al. 1988; Merson et al. 2008). Although the average of multiple repeated measures of acute status can be used to index chronic status, serial measures, such as the 24-h urine collection or hourly blood sampling, are infeasible for free-living individuals under daily life conditions, and vulnerable to loss of data and selection bias.

This study posits that it may be possible to index chronic cell hydration state, without repeat measurements, using time-lagged responses to chronic osmotic stress, which are distinct from acute responses (Yancey et al. 1982). Unlike acute cell shrinkage, chronic hyperosmotic stress upregulates metabolic pathways that favor intracellular accumulation of end products of low molecular weight (Yancey et al. 1982), and results in altered cell response to hypo-osmotic challenge (Holt et al. 1981; Evan-Wong and Davidson 1983; Arieff 1986; Kirk and Kirk 1993; Ordaz et al. 2004; Shennan and Thomson 2004).

Cell shrinkage stimulates proteolysis leading to intracellular accumulation of amino acids (Yancey et al. 1982). Cell swelling, following hypo-osmotic challenge, prompts “regulatory volume decrease,” a net efflux of osmolytes, including amino acids, out of cells (Lang et al. 1998). Cells that have adapted to chronically elevated extracellular osmolarity release more amino acids when exposed to a hypo-osmotic solution than cells maintained in isotonic solutions or cells exposed to repeated hypo-osmotic shocks (Kirk and Kirk 1993; Ordaz et al. 2004; Shennan and Thomson 2004). Given that amino acids that leave the cell can subsequently be degraded to urea in the liver (Häussinger et al. 1993; Lang et al. 1998), it is unknown if plasma concentrations or urinary excretion of amino acids can provide a window to cellular hydration state.

This study aimed to explore if a change in chronic cell hydration might be detected with single-point-in-time measures in healthy, free-living, young men who increase their “usual” level of water intake. The study focused on identifying biomarkers for individuals with “usual” low water intake, because this group is at-risk of long-term health effects of low water intake.

The study assumed that individuals with “usual” low water intake might already be adapted to chronic hyperosmotic stress at baseline. The objective was to check if recovery from this adapted state might be detected if participants were exposed to chronic hypo-osmotic conditions. The protocol aimed to induce daily hypo-osmotic exposure for 4 weeks by prescribing a daily increase in drinking water of +1 L/day relative to baseline for 2 weeks, followed by an increase of +2 L/day relative to baseline for 2 weeks. To permit inference about chronic hydration status, repeated measures of serum, urine, and saliva osmolality were used to verify whether the protocol resulted in repeated acute response to hypo-osmotic exposure. The study then tested for a decrease in red blood cell (RBC) deformability profile (an index of cell swelling) and/or decreased release of amino acids into plasma and/or urine in response to the 4-week hypo-osmotic challenge.

## Subjects and Methods

This 8-week study included healthy, normal weight men, aged 20–25 years, with 3-day mean total water intake below 2 L/day at baseline, who were similar with respect to age, gender, body weight, height, 3-day mean physical activity, dietary intake, nonsmoking, perceived stress, medication use, medical history, and laboratory indices. According to nationally representative data, ∼10% of men aged 19–50 years in the U.S. report less than 2 L/day total water intake (Institute of Medicine of the National Academies [IOM] 2004). To find seven men who met the study criteria, 349 were screened.

Participants were recruited by advertisements in local newspapers and craigslist, email listserves, and flyers posted in the community. Potential participants were screened by telephone for self-reported health status, by questionnaire for 3-day mean dietary intake and physical activity, by clinical evaluation, and by 24-h urine collection to enable completion of study measures.

The telephone exclusion criteria included a Body Mass Index (BMI) less than 18.5 or greater than 24.9 based on self-reported weight and height, weight gain or loss of greater than 2.2 kg in the previous 2 months, current smoking, a self-reported perceived stress score of 20 or higher, routine use of prescription or over the counter medicine, headache within the past 6 months, and previous physician diagnosis of high blood pressure, kidney, heart, liver or thyroid condition, glucose dysregulation, cancer, chronic pain, clinical anxiety, or depression. Other telephone exclusion criteria included inability to give informed consent in English, and a schedule that prevented weekly clinic visits and measures. Individuals who reported more than 30 min/day moderate or vigorous physical activity, or more than two alcoholic or caffeinated beverages per week on the 3-day questionnaire were excluded from participation. Individuals who were not similar to other participants with respect to 3-day mean total energy (±850 kJ/day), carbohydrate (±10% of energy), protein (±5% of energy), and sodium intake (±2000 mg/day) were excluded from the study. Clinical exclusion criteria included a measured BMI outside the normal weight range (BMI: 18.5–24.9), blood pressure over 120/80 mmHg, and any abnormal complete blood count or serum chemistry value. Individuals who did not provide a complete 24-h screening urine collection based on 24-h creatinine clearance were excluded from participation.

The protocol was approved by the Institutional Review Board of Children's Hospital & Research Center Oakland, CA. All study participants provided informed consent. Each participant was compensated each week for his participation ($1140 in total over 8 weeks). A total of seven participants completed 7 weeks of the study. Two participants did not increase water intake above baseline levels based on self-reported intake, and/or lost more than 1.5 kg body weight during the periods of higher water intake. One participant did not finish the last week of measurements due to a change in work schedule. No participant showed evidence of adverse fluid retention, that is, no increase of 3% or more body weight, increase in resting blood pressure of five or more mmHg, failure to increase urine volume, or failure to decrease urine osmolality after water loading. Data are presented for the five participants who adhered to the protocol by increasing water intake without decreasing body weight.

### Study protocol

The study had an A-B-C-A within-person design with four consecutive 14-day periods. During period 1 (weeks 1 and 2), study participants were instructed to maintain their usual diet and activity. During period 2 (weeks 3 and 4), participants were instructed to increase their total water intake to 3 L/day by consuming an additional 1 L/day (∼15 mL kg^−1^ day^−1^) drinking water. During period 3 (weeks 5 and 6), participants were instructed to increase their total water intake to 4 L/day by consuming 2 L/day (∼30 mL kg^−1^ day^−1^) drinking water. During period 4 (weeks 7 and 8), participants were instructed to decrease total water intake back to the baseline level. Participants were asked to consume the new level of drinking water every day, while continuing to drink the beverages they reported at baseline. They were supplied with bottled water during periods 2 and 3.

### Daily study protocol over 8 weeks

Except for the weekly, 2-h clinic visits, the study participants were free-living throughout the 8-week study. They were asked to maintain their usual diet, physical activity, and non-medication use. To monitor dietary intake, they were asked to keep daily 24-h records of all foods and beverages consumed during the 8 weeks. The diet record forms had free spaces to report the type, description, and amount of each food and beverage consumed. Participants were not required to weigh each item. To standardize dietary intake across the four periods, each study participant was given a copy of his week 1 diet records and asked to repeat similar intake for the following 7 weeks.

To monitor their physical activity, they were asked to wear a BodyMedia armband (Pittsburgh, PA) during waking hours every day, except while showering, swimming, or while immersed in water.

### Protocol for the weekly clinic visit

**Figure 1:**
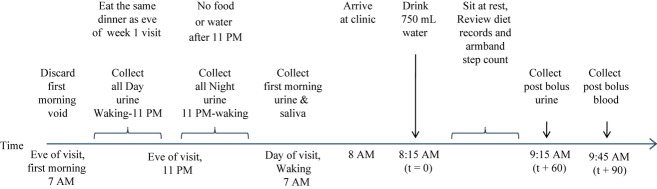
Protocol and specimen collection over 27 h, beginning on the day before the clinic visit, each week for eight consecutive weeks.

Weekly measures began on the day before the weekly clinic visit, and are described in Figure 1. On the day before the clinic visit, the participants were instructed to collect all urine after the first morning void until 11 pm in a Day urine container, all urine from 11 pm until waking the next morning in a Night container, and the first urine on the morning of the clinic visit in a first morning (FM) container. They were given labeled collection containers, a cooler, and ice packs to store and transport the urine samples. Participants were asked to consume the same dinner on the evening before their clinic visit, and restrict all food, beverage, and water intake from 11 pm until arrival at the clinic. In addition to the urine samples, they were asked to collect first morning saliva on the day of the clinic visit.

On arrival at the Children's Hospital Oakland Pediatric Clinical Research Center, the study participants emptied their bladders, if able, and consumed a bolus of 750 mL drinking water within 5 min. In the 30 min after the water bolus, study staff collected and reviewed the week's diet records and downloaded physical activity data from the BodyMedia armband. Within 1 h after the water bolus, post bolus urine was collected. Blood was collected 90 min after the water bolus.

### Dietary intake

The diet records were reviewed each week and entered into Nutrition Diet Systems (NDS-R) software (1998–2008 Nutrition Coordinating Center, University of Minnesota, Minneapolis, MN) by one certified NDS diet interviewer. The records were reviewed for completeness, and missing details were addressed to the participant during the clinic visit. The interviewer probed for beverage intake. The NDS data were used to estimate the 7-day mean daily intake of drinking water, water from other beverages, water from food, and total water (the sum of drinking water, water from other beverages, water from food, and metabolic water) for each participant. Drinking water was defined as tap, spring, mineral, or unsweetened sparkling water. Other beverages were defined as any beverage other than drinking water, following the food group codes in the NDS software. Food water was estimated as the difference between total dietary water and water from beverages. Metabolic water was estimated from the estimated protein, fat, and carbohydrate intakes, assuming that oxidation of protein, fat, and carbohydrate yield 0.41, 1.07, and 0.6 mL water/g, respectively (Buskirk and Puhl 1996). Intake of drinking water intake was expressed in absolute terms (mL) as well as relative to total water intake to index the proportion of hypo-osmotic water in the diet. Most beverages other than water have an osmolality over 285 mmol/kg, the threshold for antidiuretic hormone (ADH) release (Verbalis 2003). The NDS data were also used to estimate 7-day mean daily intake of total energy, protein, carbohydrate, sodium, and caffeine intake.

### Physical activity

Each week, the BodyMedia armband automatically recorded the hours the armband was worn, the number of steps counted, and an estimate of the active energy expenditure during the time the armband was worn. The armband estimates active energy expenditure using proprietary equations developed by the manufacturer that have been validated by indirect open-circuit calorimetry during various types of exercise (walking, cycling, stepping, and arm ergometry) in healthy, normal weight subjects (Jakicic et al. 2004). Armband estimates for a given week were excluded from the analysis if the armband was worn for less than 12 h during the week. Four participants wore the armband for at least 60 h (up to 144 h) at baseline. One participant had technical difficulties with his armband at baseline. All five participants wore the armband for at least 35 h (up to 168 h) in period 2 and at least 60 h (up to 152 h) in period 3.

### Urine collection and laboratory tests

Urine volume, creatinine (ARUP Laboratories, Salt Lake City, UT), and osmolality were determined on fresh urine. All osmolality measurements were made in triplicate using a freezing point depression osmometer (Advanced Instruments, Norwood, MA). The FM–Post bolus difference in urine osmolality was calculated to check for protocol (bolus)-induced cell swelling. Completeness of the 24-h urine collection was checked using 24-h creatinine excretion (Landry and Bazari 2011). Urine aliquots were stored frozen at −80°C until sent to ARUP laboratories for determination of the Day-urine ADH concentration and the post bolus urine amino acid content. The urine nitrate and nitrite concentration of the Post Bolus urine was determined with a colorimetric assay kit (Cayman Chemical Company, Ann Arbor, MI) (Nims et al. 1995).

### Saliva collection and laboratory tests

Each week, for the measurement of biologically active free cortisol (Raff 2009), study participants passively collected saliva by not swallowing for 1–2 min and drooling through a straw. The samples were transported on ice to the clinic, stored at −80°C, and assayed for saliva cortisol using a commercially available ELISA kit (IBL International Corp., Toronto, Ontario, Canada).

### Blood collection and laboratory tests

Each week, 60 mL fasting blood was collected 90 min after the 750 mL water bolus. Fresh, refrigerated, K_2_EDTA-anticoagulated whole blood was used to determine the RBC, mean corpuscular hemoglobin concentration (MCHC), and reticulocyte count by automated cell counter (Advia 120; Bayer Healthcare, Tarrytown, NY). Plasma separated from K_2_EDTA-anticoagulated blood was stored frozen at −80°C, and sent to ARUP Laboratories for plasma amino acid determination.

Serum osmolality was determined on fresh serum by freezing point depression osmometer. Serum aliquots were stored frozen at −80°C until sent to Quest Diagnostics (San Jose, CA) for determination of serum glucose, serum urea nitrogen (BUN), and serum BUN:creatinine ratio. Serum insulin was determined by ELISA using a commercially available kit (IBL International Corp.). The Homeostasis Model Assessment (HOMA) index of insulin resistance, validated by hyperglycemic and euglycemic clamp, was calculated using the formula described by Matthews et al. (1985): insulin (μU/m) × [glucose (mmol/L)/22.5].

### Red blood cell deformability

**Figure 2:**
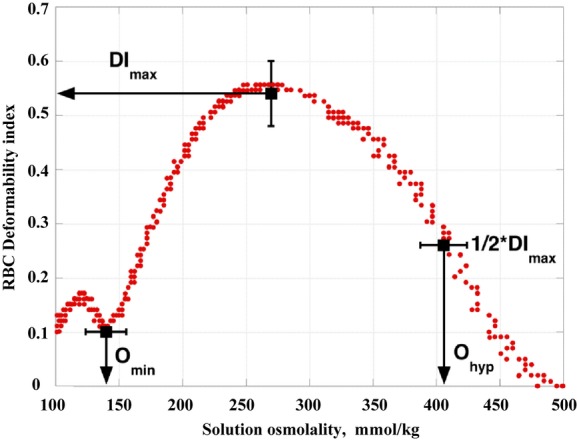
Osmotic deformability profile of red blood cells (RBC) by ektacytometry. A typical osmotic deformability curve indicating the points used for comparison RBC deformation; The osmolality where minimal deformability is found (Omin), the maximum deformability (DImax), and the osmolality where the deformability is half of DImax at high osmolality (Ohyp).

RBC deformability as measured by ektacytometry varies with cellular water content (Clark et al. 1983). Dextrose-anticoagulated whole blood was suspended in a viscometer and exposed to shear stress in solutions ranging in osmolality from hypotonic to hypertonic by a sodium chloride gradient (RBC Lab, CHORI, Oakland, CA). The osmotic deformability profile was defined by three points as indicated in Figure 2.

### Statistics

Stata software (Stata SE, version 9.2; StataCorp, College Station, TX) was used for all analyses. For each variable studied, within-person change (fixed effects) models that allow each participant to serve as his own control, were used to test for statistically significant change over time relative to the 2-week baseline period. Each parameter was the dependent variable in the model with time as independent variable. To account for the U-shaped intervention design (A-B-C-A), time was represented either as a set of dummy variables (one for each period or week), or as continuous time with a timextime interaction term. Time was expressed as a continuous variable to test for linear trends across periods A-B-C. Given the small sample size and multiple parameters tested, the analysis goal was to only identify variables that showed both a statistically significant U-shaped change in the fixed effect model (*P < 0.05 for the main effect and interaction term) as well as a consistent U-shaped pattern of change across all five participants. To check for change in the whole distribution of RBC deformability, fixed effect models were also used to test for correlated change over time between ektacytometry variables that index different aspects of the same distribution.

## Results

### Sample characteristics

Table 1 describes the weight and height of each study participant. The mean ± SEM body weight and height of the study participants was 66 ± 2 kg and 168 ± 1 cm. Table 2 shows 7-day mean daily nutrient intake by study week. Over the 8-week study, there were no significant systematic changes in total energy or macronutrient intake, or physical activity as assessed by step count. Caffeine intake was below 20 mg/day throughout the 8-week study. Except for one participant who reported 15 g of alcohol on 1 day in week 4, the participants did not report consuming alcoholic beverages.

**Table 1:** Baseline weight and height of each study participant

	Mean ± SEM	Participant ID				

		1	2	3	4	5
Weight, kg	66 ± 2	63.1	69.2	66.4	69.7	62.1
Height, cm	168 ± 1	167.7	169.0	170.1	168.1	165.6

**Table 2:** Dietary intake^1^ and active energy expenditure^2^ of healthy young men who incrementally increased water intake over two 2-week periods^3^

	Baseline (total water<2 L/day)	Period 2 (+1 L/day drinking water)		Period 3 (+2 L/day drinking water)		Period 4 (return to baseline)	
				
	Week 1–2	Week 3	Week 4	Week 5	Week 6	Week 7	Week 8
Drinking water, mL/day	398 ± 168	1208 ± 213*	1244 ± 150*	1975 ± 273*	1945 ± 183*	1096 ± 469*	427 ± 57**
Drinking water, % of total water	17 ± 4	46 ± 4*	48 ± 4*	61 ± 2*	61 ± 3*	37 ± 8*	25 ± 2*
Water from other beverages, mL/day	635 ± 171	463 ± 142	433 ± 148*	479 ± 252*	374 ± 123*	432 ± 223*	411 ± 147*
Water from food, mL/day	815 ± 162	698 ± 110	671 ± 122	641 ± 98	694 ± 156	742 ± 154	631 ± 102
Metabolic water, mL/day	268 ± 26	256 ± 18	259 ± 9	206 ± 17*	254 ± 15	236 ± 22	235 ± 11
Total water, mL/day	2117 ± 463	2625 ± 403	2607 ± 281	3300 ± 570*	3266 ± 427*	2506 ± 760	1705 ± 177**
Total energy, kJ/day	8439 ± 770	8088 ± 594	8284 ± 297	6623 ± 527	8033 ± 544	7519 ± 699	7448 ± 331
Total protein, g/day	90 ± 8	76 ± 8	86 ± 9	70 ± 6	80 ± 11	82 ± 11	72 ± 7
Total carbohydrate, g/day	237 ± 20	223 ± 15	213 ± 12	171 ± 18*	218 ± 17	208 ± 26	206 ± 16
Sodium, mg/day	3737 ± 274	3758 ± 588	3954 ± 564	3391 ± 571	3414 ± 328	3687 ± 322	3294 ± 523
Caffeine, mg/day	20 ± 10	10 ± 6	18 ± 6	4 ± 3*	4 ± 2*	6 ± 4*	9 ± 4
Steps, number/day	8426 ± 2326	7663 ± 2232	7254 ± 1679	7294 ± 1277	6785 ± 969	7692 ± 1599	–
Armband time, h/week	72 ± 21	77 ± 31	78 ± 28	92 ± 21	69 ± 21	108 ± 37	–
Active EE, kJ/h	50 ± 8	50 ± 13	71 ± 38	33 ± 4	46 ± 13	29 ± 4	–

1 The 7-day mean daily water and nutrient intakes were estimated from 24-h diet records using NDS software. Total water includes drinking water, water from other beverages, food and metabolic water.

2 The mean steps and active energy expenditure were estimated by a BodyMedia armband worn over more than 35 h each week.

3 Data are presented as mean ± SEM, *n* = 5, except for week 8 where *n* = 4.

* Significantly different from the baseline value, *P* < 0.05.

** Significant U-shaped change over the 8 weeks, *P* < 0.05.

### Water intake

Intake of drinking water changed following a U-shaped pattern for all five participants. Intake of drinking water was significantly greater relative to baseline during weeks 3 through 7. In period 2 (weeks 3 and 4), the mean ± SEM increase in drinking water was +0.8 ± 0.3 L/day. In period 3 (weeks 5 and 6), the mean ± SEM increase was +1.5 ± 0.3 L/day. The increase in drinking water exceeded 1 L/day for all five participants. In week 8, intake of drinking water did not differ from baseline values.

Although water intake from food did not vary significantly over time, intake of other beverages was significantly decreased in weeks 3 through 6 (mean ± SEM: −0.2 ± 0.06 L/day), which partially offset the increases in drinking water. Mean ± SEM total water intake did not differ significantly from baseline in period 2, but was 1.1 ± 0.3 L/day higher than baseline in period 3. Expressed in relative terms, the increase in drinking water tripled the proportion of hypotonic water in the diet from less than 20% in period 1 to 61% in period 3.

### Chronic cell hydration as indexed by repeated acute measures

#### Serum osmolality

Serum osmolality was significantly lower in period 3 relative to baseline, although the U-shaped trend did not reach statistical significance over the 8 weeks (see Table 3). A decrease of more than 5 mmol/kg (290–284 mmol/kg) was observed for two participants.

**Table 3:** Chronic cell hydration status of healthy young men who incrementally increased water intake over two 2-week periods, as indexed by aggregated serial indices of acute status^1^

	Baseline (total water<2 L/day)	Period 2 (+1 L/day drinking water)	Period 3 (+2 L/day drinking water)	Period 4 (return to baseline)
	Weeks 1 and 2	Weeks 3 and 4	Weeks 5 and 6	Weeks 7 and 8
Post bolus serum osmolality, mmol/kg^2^	289 ± 1	288 ± 1	287 ± 1*	289 ± 1
Urine concentration^3^,^4^				
Day urine ADH, pmol/L	11 ± 2	7 ± 2	5 ± 2*	9 ± 3**
Day urine osmolality, mmol/kg	617 ± 87	472 ± 83	303 ± 73*	634 ± 99**
Complete day urine osmolality, mmol/kg	575 ± 125	507 ± 101	397 ± 106*	661 ± 94**
Complete 24-h urine osmolality, mmol/kg	591 ± 128	441 ± 55	352 ± 50*	594 ± 98**
Complete 24-h urine volume, mL	1474 ± 280	1724 ± 262	2608 ± 230*	1327 ± 239**
Insulin resistance				
Post bolus serum glucose, mmol/L^2^	4.4 ± 0.1	4.5 ± 0.1	4.4 ± 0.1	4.6 ± 0.1
Post bolus serum insulin, pmol/L^2^	67.8 ± 6.6	57.6 ± 5.4	51.6 ± 4.2*	61.2 ± 4.8
Post bolus HOMA^5^	2.2 ± 0.2	1.9 ± 0.2	1.7 ± 0.1*	2.1 ± 0.2**
Stress response				
First morning saliva cortisol, nmol/L	29.0 ± 6.9	20.7 ± 4.7	17.7 ± 4.7*	22.1 ± 3.6

1 The data presented represent the mean ± SEM of 2 weekly measurements per period per participant, *n* = 5 except for week 8 where *n* = 4.

2 The serum indices were determined from blood collected 90 min after a 750 mL bolus of drinking water, following overnight water and food restriction.

3 The Day urine indices were determined from urine collected on the day before each clinic visit, after waking until 11 pm. The Day mean values include all time points for all participants. The same pattern of results is observed when the analysis is restricted to Day urine samples from complete 24-h collections only.

4 The 24-h parameters were estimated for collections identified as complete based on 24-h creatinine clearance. 70% of the 24-h urine collections were complete.

5 HOMA: Homeostasis Model Assessment index (Matthews et al. 1985).

* Significantly different compared to the corresponding value at baseline, *P* < 0.05.

** Significant U-shaped change over the four study periods, *P* < 0.05.

#### Urine ADH and urine osmolality

Change in urine ADH and urine osmolality followed a U-shaped pattern for all five participants (see Table 3). The Day-urine ADH and Day-urine osmolality were significantly decreased relative to baseline in period 3, with mean ± SEM decreases of 6.5 ± 2.8 pmol/L and 313 ± 101 mmol/kg, respectively. The same pattern of results was observed when analysis was restricted to Day-urine samples from complete collections only. Based on 24-h creatinine clearance, only 27 out of 39 24-h urine collections (70%) were complete. Among complete collections, the mean ± SEM 24-h urine osmolality was 329 ± 122 mmol/kg lower in period 3 compared to baseline. The mean ± SEM Day and 24-h urine osmolality were both below 500 mmol/kg in period 3. The 24-h urine volume was significantly increased relative to baseline in period 3 by an estimated 1000 ± 400 mL/day.

#### Insulin resistance

Change in HOMA followed a U-shaped pattern for four out of five participants. In period 3, the HOMA index of insulin resistance was significantly lower than baseline. The 2-week mean fasting glucose did not differ by study period.

#### Cortisol

First morning saliva cortisol was lower in period 3 relative to baseline for four out of five participants, although the U-shaped trend did not reach statistical significance.

### 750 mL water bolus as hypo-osmotic stimulus

**Figure 3:**
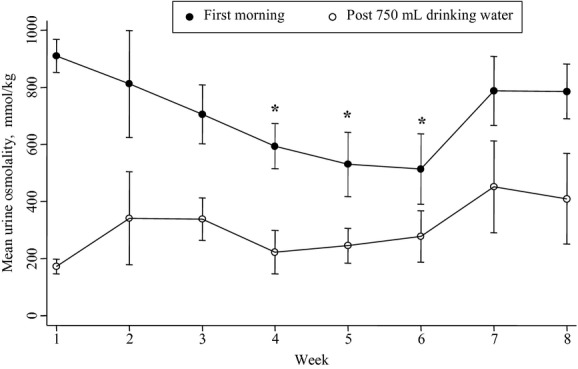
Urine osmolality of healthy young men who incrementally increased water intake over two 2-week periods. The data presented are mean ± SEM, *n* = 5 except for week 8 where *n* = 4. Each week for 8 weeks, urine was collected before (•) and 60 min after (○) a 750 mL bolus of drinking water, following overnight water and food restriction. *Significantly different from the baseline value (mean of weeks 1 and 2), *P* < 0.05. The change in first morning urine osmolality over the 8 weeks was significantly U-shaped (*P* < 0.05).

Each week, study participants were given a 750 mL bolus of drinking water as hypo-osmotic stimulus following overnight water restriction. Figure 3 describes the mean ± SEM urine osmolality before and after this bolus. Despite significantly lower first morning urine osmolality relative to baseline in period 2 (mean ± SEM: −217 ± 74 mmol/kg, *P* = 0.007) and period 3 (−345 ± 74 mmol/kg, *P* < 0.0001), the water bolus resulted in urine dilution for all five participants in all weeks, with only one exception, when the first morning urine osmolality was already below 100 mmol/kg.

### Potential single-point-in-time indices of chronic cell hydration status

#### Downward shift in RBC deformability profile

**Figure 4:**
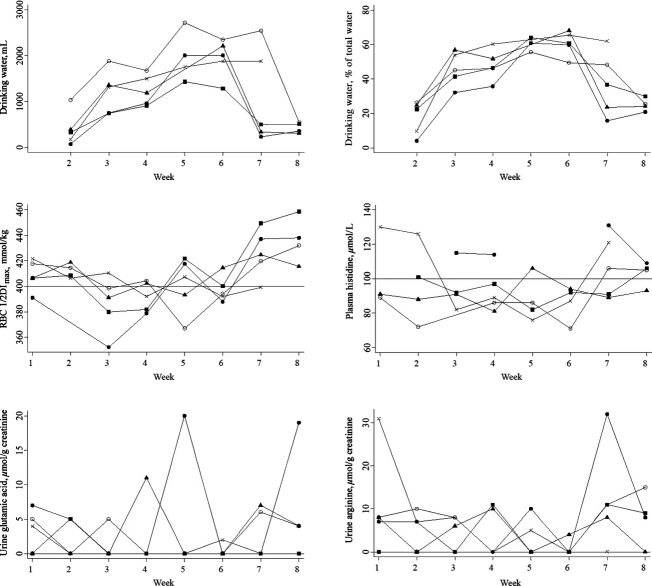
Change in intake of drinking water, RBC deformability, post bolus plasma histidine, post bolus urine glutamic acid, and post bolus urine arginine for each study participant. Ohyp: The osmolality where the RBC deformability is half of DImax at high osmolality (see Fig. 2). Each week for 8 weeks, amino acid concentrations were determined from urine and plasma collected after a 750 mL bolus of drinking water, following overnight water and food restriction. The dotted lines illustrate the arbitrarily chosen cutoffs for classifying participants with respect to Ohyp over 400 mmol/kg, plasma histidine over 100 μmol/L, and undetectable urine glutamic acid and urine arginine.

Table 4 describes the range of the RBC deformability profile as observed each week. In period 1, at baseline, the mean ± SE of Omin was 139 ± 2 mmol/kg. The mean ± SE of Ohyp was 410 ± 3 mmol/kg. Beginning in week 3, the whole distribution shifted to the left. The mean ± SE of Omin was 137 ± 3 mmol/kg. The mean ± SE of Ohyp was 394 ± 4 mmol/kg. The Ohyp was under 400 mmol/kg during the weeks of higher water intake for four out of five participants, and over 400 mmol/kg in period 4 when participants returned to their baseline levels of water intake. The pattern of change over 8 weeks in Omin was significantly U-shaped. The pattern of change in Ohyp, was also significantly U-shaped. The changes in Omin and Ohyp were significantly associated in within-person (fixed effect) change models (*P* < 0.000). A 1 mmol/kg change in Omin was associated with a 3 (0.3) mmol/kg change in Ohyp. Figure 4 describes Ohyp over the 8 weeks for each participant.

**Table 4:** Red blood cell (RBC) osmotic deformability of healthy young men who incrementally increased water intake over two 2-week periods^1^,^2^

	Baseline (total water intake<2 L/day)		Period 2 (+1 L/day drinking water)		Period 3 (+2 L/day drinking water)		Period 4 (return to baseline)	
				
	Week 1	Week 2	Week 3	Week 4	Week 5	Week 6	Week 7	Week 8
RBC Omin, mmol/kg^3^	140 ± 3	137 ± 3	138 ± 2	136 ± 2	138 ± 5	136 ± 3	147 ± 5*	150 ± 3*,**
RBC Ohyp, mmol/kg^4^	409 ± 5	412 ± 3	386 ± 10*	392 ± 5	401 ± 10	398 ± 5	426 ± 8	436 ± 9*,**
Ohyp-Omin, mmol/kg	268 ± 7	275 ± 5	249 ± 10*	255 ± 6	264 ± 7	261 ± 5	279 ± 5	286 ± 6*,**
RBC DImax^5^	0.52 ± 0.02	0.53 ± 0.01	0.51 ± 0.03	0.51 ± 0.01	0.52 ± 0.02	0.54 ± 0.02	0.57 ± 0.01*	0.55 ± 0.02***
Reticulocyte,%	1.2 ± 0.1	1.1 ± 0.3	1.5 ± 0.1	1.4 ± 0.1	1.5 ± 0.1*	1.4 ± 0.1	1.4 ± 0.1*	1.7 ± 0.2*,***
Reticulocyte, number	64.3 ± 4.8	55.8 ± 17.4	73.8 ± 4.3	63.6 ± 5.5	74.1 ± 5.0	71.2 ± 7.1	70.6 ± 7.2	85.9 ± 10.3*
MCHC, g/dL	35.8 ± 0.3	35.1 ± 0.3	35.4 ± 0.3	34.8 ± 0.9	35.9 ± 0.2	35.5 ± 0.2	34.9 ± 0.4	35.9 ± 0.1

1 The data presented are mean ± SEM, *n* = 5 except for week 8 where *n* = 4.

2 The RBC deformability was determined from blood collected 90 min after a 750 mL bolus of drinking water, following overnight water and food restriction. The RBC osmotic deformability profile was defined by three points as indicated in Figure 2:

3 Omin: the osmolality where minimal deformability was found.

4 Ohyp: the osmolality where the deformability is half of DImax at high osmolality.

5 DImax: the maximum deformability around isotonicity.

* Significantly different compared to the corresponding value at baseline, *P* < 0.05.

** Significant U-shaped change over the four study periods, *P* < 0.05.

*** Significant linear trend over the four study periods, *P* < 0.05.

#### Decreased plasma and urine amino acid efflux

**Figure 5:**
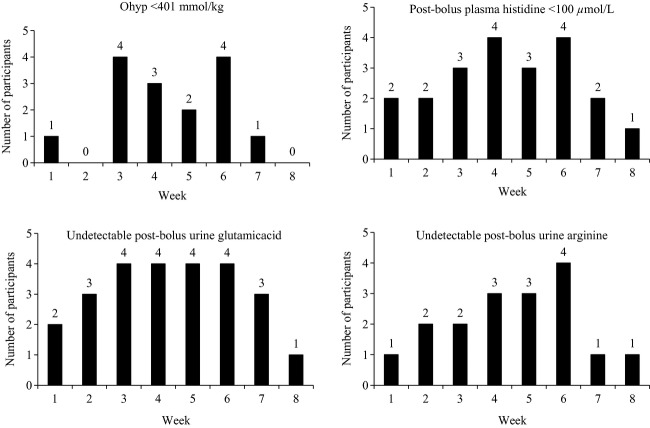
Number of participants with U-shaped change in RBC deformability, post bolus plasma histidine, post bolus urine glutamic acid, and post bolus urine arginine, associated with incremental increases in water intake over two 2-week periods. Ohyp: The osmolality where the RBC deformability is half of DImax at high osmolality (see Fig. 2). Each week for 8 weeks, amino acid concentrations were determined from urine and plasma collected after a 750 mL bolus of drinking water, following overnight water and food restriction.

Table 5 describes the plasma amino acid concentrations each week, 90 min after the participants consumed the 750 mL water bolus. A U-shaped pattern, with lower plasma amino acid concentrations during the periods of higher water intake, was observed for plasma histidine. Plasma histidine was below 100 μmol/L during the periods of higher water intake for four participants (see Figs. 4 and 5). The total sum of plasma amino acids and serum BUN did not vary significantly over the 8 weeks.

**Table 5:** Post bolus plasma amino acid (AA) concentrations of healthy young men who incrementally increased water intake over two 2-week periods^1^,^2^

	Baseline (<2 L/day total water intake)		Period 2 (+1 L/day drinking water)		Period 3 (+2 L/day drinking water)		Period 4 (return to baseline)	
				
	Week 1 (*n* = 3)	Week 2 (*n* = 4)	Week 3 (*n* = 5)	Week 4 (*n* = 5)	Week 5 (*n* = 4)	Week 6 (*n* = 4)	Week 7 (*n* = 5)	Week 8 (*n* = 4)
μmol/L								
Alanine	254 ± 24	267 ± 32	282 ± 25	293 ± 17	254 ± 11	288 ± 20	296 ± 22	300 ± 36
Arginine	81 ± 3	92 ± 7	86 ± 6	87 ± 6	83 ± 2	99 ± 8	89 ± 5	94 ± 9
Aspartic acid	5 ± 2	4 ± 0	4 ± 0	5 ± 1	6 ± 2	5 ± 0	6 ± 1	6 ± 2*
Citrulline	30 ± 3	33 ± 2	34 ± 3	32 ± 2	33 ± 2	34 ± 3	33 ± 2	33 ± 3
Cystine	24 ± 5	32 ± 2	30 ± 4	35 ± 3	34 ± 2	35 ± 3	34 ± 3	34 ± 1*
Glutamic acid	20 ± 4	19 ± 1	23 ± 6	21 ± 4	21 ± 3	28 ± 5**	27 ± 6**	22 ± 3*
Glutamine	535 ± 18	547 ± 14	550 ± 18	569 ± 7	544 ± 19	560 ± 25	563 ± 13	558 ± 23
Glycine	220 ± 12	240 ± 16	207 ± 14	231 ± 26	218 ± 10	251 ± 14	238 ± 21	226 ± 13
Histidine	103 ± 13	97 ± 11	95 ± 7	93 ± 6	88 ± 7	86 ± 5	108 ± 8	103 ± 4***
Hydroxyproline	6 ± 6	5 ± 5	1 ± 1	6 ± 4	8 ± 8	8 ± 5	3 ± 3	4 ± 2
Isoleucine	51 ± 3	59 ± 7	65 ± 5	59 ± 5	66 ± 7**	60 ± 5	57 ± 4	56 ± 2***
Leucine	117 ± 9	121 ± 8	120 ± 4	122 ± 6	129 ± 10	119 ± 9	122 ± 6	120 ± 4
Lysine	177 ± 13	172 ± 11	163 ± 10	184 ± 8	172 ± 16	171 ± 11	184 ± 11	179 ± 12
Methionine	21 ± 3	20 ± 3	22 ± 2	22 ± 1	23 ± 2	23 ± 3	24 ± 1	21 ± 1
Ornithine	46 ± 6	47 ± 4	45 ± 3	52 ± 7	48 ± 5	49 ± 4	44 ± 2	45 ± 2
Phenylalanine	48 ± 4	49 ± 2	51 ± 3	50 ± 2	47 ± 1	50 ± 4	53 ± 2	50 ± 2
Proline	142 ± 2	159 ± 9	145 ± 6	142 ± 11	129 ± 7	159 ± 3	161 ± 11	154 ± 16
Serine	102 ± 5	93 ± 8	82 ± 6	97 ± 10	92 ± 9	103 ± 7	99 ± 7	88 ± 4
Taurine	45 ± 4	46 ± 4	50 ± 5	48 ± 4	44 ± 2	47 ± 3	45 ± 4	51 ± 4
Threonine	110 ± 9	106 ± 15	109 ± 7	117 ± 9	112 ± 11	125 ± 12**	116 ± 2	105 ± 6
Tyrosine	43 ± 2	48 ± 3	49 ± 4	49 ± 2	45 ± 2	49 ± 2	52 ± 2**	52 ± 2*,**
Valine	215 ± 12	203 ± 17	204 ± 6	216 ± 10	218 ± 20	201 ± 16	201 ± 9	208 ± 10
Sum of AA^3^	2394 ± 86	2458 ± 128	2462 ± 25	2529 ± 85	2411 ± 79	2545 ± 83	2571 ± 95	2497 ± 90
Arginine:methionine	4.2 ± 0.8	4.8 ± 0.6	4.0 ± 0.2	3.7 ± 0.3	3.7 ± 0.3**	4.5 ± 0.5	3.8 ± 0.3	4.5 ± 0.3
Glutamate:methionine	1.1 ± 0.4	1.0 ± 0.2	1.1 ± 0.3	1.0 ± 0.2	0.9 ± 0.1	1.3 ± 0.3	1.1 ± 0.3	1.1 ± 0.1
Glutamine:glutamate	29 ± 6	30 ± 2	33 ± 13	30 ± 4	28 ± 3	22 ± 4	26 ± 6	27 ± 4*
Serum creatinine	72 ± 4	72 ± 4	73 ± 4	71 ± 4	72 ±	72 ± 5	71 ± 4	73 ± 5
BUN: creatinine ratio	17.7 ± 1.0	16.9 ± 1.9	16.7 ± 0.7	17.2 ± 1.2	17.1 ± 1.8	15.0 ± 1.9	17.9 ± 1.9	18.7 ± 3.2
mmol/L								
Serum BUN	4.9 ± 0.2	4.9 ± 0.5	4.9 ± 0.2	4.9 ± 0.4	5.1 ± 0.6	4.4 ± 0.7	5.1 ± 0.5	5.4 ± 0.7

1 The data presented are mean ± SEM. Plasma was not available for amino acid determination for all participants at all time points.

2 The amino acid concentrations were determined from blood collected 90 min after a 750 mL bolus of drinking water, following overnight water and food restriction.

3 The sum of amino acid (AA) includes all of the amino acids tested.

* Significant linear trend over the 8 weeks, *P* < 0.05.

** Significantly different from the baseline value (mean of weeks 1 and 2), *P* < 0.05.

*** Significant U-shaped change over the four study periods, *P* < 0.05.

Table 6 describes the urine amino acid concentrations each week, within 60 min after the participants consumed 750 mL drinking water. A U-shaped pattern of reduced efflux was observed for urine arginine and urine glutamic acid during the periods of higher water intake. Both urine arginine and urine glutamic acid decreased below detection limits for four out of five participants (see Figs. 4 and 5). Other urine amino acid concentrations did not decrease significantly over time. The sum of urine amino acids did not vary significantly over time. The post bolus urine nitrate and nitrite concentration did not vary significantly over time.

**Table 6:** Post bolus urine amino acid (AA) concentrations of healthy young men who incrementally increased water intake over two 2-week periods^1^,^2^

	Baseline (<2 L/day total water intake)		Period 2 (+1 L/day drinking water)		Period 3 (+2 L/day drinking water)		Period 4 (return to baseline)	
				
	Week 1	Week 2	Week 3	Week 4	Week 5	Week 6	Week 7	Week 8
μmol/g creatinine								
Creatinine	29 ± 5	53 ± 23	62 ± 23	51 ± 19	37 ± 10	64 ± 26	92 ± 37*	63 ± 33
Alanine	112 ± 9	156 ± 22	128 ± 23	168 ± 23	150 ± 21	170 ± 23	150 ± 24	150 ± 19
Arginine	11 ± 5	5 ± 2	4 ± 2	4 ± 3	3 ± 2	1 ± 1	12 ± 5	8 ± 3**
Aspartic acid	112 ± 16	115 ± 8	108 ± 8	111 ± 7	130 ± 7	121 ± 4	99 ± 12	115 ± 12
Citrulline	3 ± 1	4 ± 2	3 ± 1	3 ± 1	6 ± 3	9 ± 5	14 ± 4*	12 ± 4*,***
Cystine	32 ± 2	34 ± 4	32 ± 4	36 ± 4	38 ± 5	31 ± 2	33 ± 4	33 ± 3
Glutamic acid	3 ± 1	2 ± 1	1 ± 1	2 ± 2	4 ± 4	0 ± 0	3 ± 2	7 ± 4**
Glutamine	223 ± 17	279 ± 22	235 ± 17	299 ± 49	299 ± 15	257 ± 35	290 ± 28	264 ± 25
Glycine	662 ± 78	853 ± 149	639 ± 81	820 ± 112	758 ± 104	772 ± 174	799 ± 122	825 ± 87
Histidine	357 ± 33	409 ± 37	357 ± 53	481 ± 99	485 ± 70	409 ± 86	482 ± 70	420 ± 88
Isoleucine	4 ± 1	7 ± 3	4 ± 1	6 ± 2	5 ± 1	4 ± 1	3 ± 2	3 ± 2
Leucine	37 ± 10	33 ± 8	38 ± 7	36 ± 9	28 ± 11	30 ± 9	42 ± 7	49 ± 6
Lysine	123 ± 20	166 ± 20	135 ± 17	170 ± 35	172 ± 36	146 ± 21	155 ± 23	180 ± 42*,***
Methionine	18 ± 3	21 ± 4	20 ± 3	26 ± 4	30 ± 6*	22 ± 5	16 ± 1	14 ± 2**
Ornithine	9 ± 3	10 ± 5	8 ± 5	7 ± 3	8 ± 1	6 ± 0	5 ± 1	4 ± 2***
Phenylalanine	31 ± 2	35 ± 4	33 ± 4	40 ± 6	37 ± 3	32 ± 4	38 ± 4	39 ± 3
Serine	198 ± 29	219 ± 27	199 ± 29	244 ± 56	227 ± 30	214 ± 30	224 ± 26	210 ± 32
Taurine	1038 ± 233	1039 ± 324	1003 ± 376	1277 ± 508	1154 ± 432	1617 ± 589*	1145 ± 434	1290 ± 560
Threonine	59 ± 2	74 ± 11	59 ± 7	94 ± 23*	80 ± 10	80 ± 8	83 ± 10	66 ± 5
Tyrosine	47 ± 6	55 ± 10	50 ± 9	56 ± 11	56 ± 6	44 ± 6	54 ± 6	50 ± 6
Valine	20 ± 1	22 ± 3	19 ± 3	23 ± 2	21 ± 3	17 ± 3	21 ± 2	18 ± 4
Sum of AA^3^	84 ± 12	192 ± 94	181 ± 68	154 ± 46	114 ± 19	178 ± 58	262 ± 86	177 ± 65
Arginine:methionine	0.6 ± 0.3	0.3 ± 0.1	0.3 ± 0.1	0.2 ± 0.2	0.1 ± 0.1	0.0 ± 0.0	0.8 ± 0.3	0.6 ± 0.2**
Glutamate:methionine	0.2 ± 0.1	0.1 ± 0.1	0.1 ± 0.1	0.2 ± 0.	0.2 ± 0.2	0.0 ± 0.0	0.1 ± 0.1	0.6 ± 0.5
mmol/L								
Urine nitrate+nitrite	117 ± 24	264 ± 61	241 ± 84	213 ± 80	187 ± 77	207 ± 86	335 ± 128	270 ± 114

1 The data presented are mean ± SEM, *n* = 5 except for week 8 where *n* = 4.

2 The amino acid (AA) concentrations were determined from urine collected 60 min after a 750 mL bolus of drinking water, following overnight water and food restriction.

3 The sum of AA includes all of the amino acids tested.

* Significantly different from the baseline value (mean of weeks 1 and 2), *P* < 0.05.

** Significant U-shaped change over the four study periods, *P* < 0.05.

*** Significant linear trend over the 8 weeks, *P* < 0.05.

## Discussion

The results of this study suggest that it may be possible to index chronic cell hydration status in healthy, free-living individuals with biomarkers collected at a single-point-in-time, as opposed to repeated assessment of acute status. The study identified four parameters to pursue as possible biomarkers of chronic cell hydration status.

### Protocol adherence

The 8-week protocol induced significant U-shaped change in total water intake, hypo-osmotic water (plain water as a percent of total water) intake, and 2-week cell hydration status over four consecutive 2-week periods, despite significant decreases in other beverage intake. Aggregate, serial assessment of *acute* cell hydration status indicated change in *chronic* cell hydration status. The periods of higher water intake were associated with decreased 2-week mean serum osmolality and decreased compensation for hyperosmotic stress on cells (Bratusch-Marrain and DeFronzo 1983; Star 1990; Berneis et al. 1999), as reflected by lower Day-urine ADH, 24-h urine osmolality, and 2-week mean HOMA index, as well as greater 24-h urine volume. A consistent pattern of change was observed for all five participants.

Each week, one blood and one urine sample were collected after the participants ingested 750 mL drinking water, following overnight food and water restriction. Each week, the water bolus was associated with urine dilution, suggesting that the hypo-osmotic challenge was adequate to swell cells and inhibit ADH release (Star 1990; Greenleaf 1992). The stimulus was effective for all five participants.

### Possible biomarkers of chronic cell hydration obtained from one sample of blood or urine

To address the problem of multiple statistical tests, the analysis identified parameters that met two criteria to pursue in future studies: a significant U-shaped pattern of change over the 8-week study, as well as a consistent pattern of change across participants. Four parameters were identified that met these criteria. All four parameters are plausible biomarkers of chronic cell hydration status, given their relationships with cell hydration (Chien 1977; Clark et al. 1983; Leaf et al. 1989; Kjaer et al. 1995; Chaplin 2012; Tousoulis et al. 2012).

Ektacytometry variables derived from the post bolus blood sample varied over the 8-week study following a U-shaped pattern that was consistent across participants. The Omin and Ohyp, which respectively delineate the osmolality where minimal RBC deformability is found and the osmolality where RBC deformability is half of DImax, both decreased, indicating a shift in the whole distribution of RBC deformability down, over the range of osmolality during the periods of higher water intake. The correlated decrease is consistent with reduced cell water associated with reduced intracellular solute (Clark et al. 1983), during the periods of higher water intake. If the observed changes reflect true change in the whole distribution and/or change in intracellular solute, then other indicators of the RBC deformability distribution, besides Ohyp, and/or intracellular solute, might also be plausible indicators of chronic cell hydration status,

In this study, the Ohyp showed greater variability than Omin. The Ohyp decreased 3 mmol/kg for each 1 mmol/kg decrease in Omin. The disproportionate changes initially resulted in a narrower range of RBC deformability in the first week of higher water intake, followed by a progressively wider deformability range through week 8, which was significantly associated with an increase in DImax in weeks 7 and 8. The data are consistent with results from Clark et al. (1983), where Nystatin-induced variation in normal RBC water content unequally displaced the RBC deformability profile along the osmotic axis on either side of the maximum DI. High-water-content cells deformed over a broader osmotic range than low-water-content cells. Clark et al. (1983) explain that, unlike Ohyp, which is primarily determined by cell water content, the Omin and DImax depend on cell membrane area as well as water content. It is important to note that the maximum DI depends on instrumental variables. Differences in DImax between studies may reflect different instrumental conditions. All measurements in this study were made under the same instrumental conditions, making within-study comparisons valid.

The present data suggested a cutoff of 400 mmol/kg to classify participants with respect to chronic cell hydration state using Ohyp. The cutoff was arbitrarily chosen. It remains to be confirmed whether Ohyp values over 400 mmol/kg mean higher RBC intracellular solute content than values of 400 mmol/kg or lower.

Plasma histidine concentrations were significantly decreased during the periods of higher water intake. Given no parallel significant change in urine histidine, relative to creatinine, the change in plasma histidine was likely not attributable to increased renal clearance, dietary histidine deficiency, or malabsoprtion. It remains to be determined if the change reflects decreased histidine release into plasma and/or increased cellular uptake of histidine. Consistent with other studies (Nair et al. 1995; Stancáková et al. 2012), decreases in plasma histidine were observed with decreases in insulin and increases in plasma isoleucine. The increase in plasma isoleucine could indicate increased muscle protein breakdown, that is, less insulin suppression of proteolysis, as well as increased insulin-independent muscle glucose uptake (Doi et al. 2007). Increases in protein breakdown due to decreased insulin concentrations might have countered decreases in protein breakdown stimulated by cell-swelling, explaining the lack of change in plasma urea, as well as the stable plasma glucose.

Two parameters determined from the post bolus urine sample varied over the 8 weeks following a U-shaped pattern that was consistent across participants. Both urine arginine and glutamic acid decreased below detection limits during the periods of higher water intake.

The extent of change in urine arginine and glutamate concentration could not be explained by urine dilution alone. There was significantly less release of these amino acids, relative to release of creatinine, in response to ingestion of 750 mL drinking water.

The change in urine arginine excretion was not associated with a decrease in plasma arginine. The decrease did not appear attributable to increased NO synthesis, as the urine nitrate/nitrite concentration did not change over time. A decrease in renal synthesis of arginine from glutamate cannot be ruled out. Plasma citrulline, a limiting factor for renal arginine synthesis, was not decreased (Dhanakoti et al. 1990; Chen and Baylis 2010).

Plasma glutamate is tightly regulated by transport into cells and renal clearance (Raj et al. 2001). The significant decrease in urine glutamate was associated with a significant increase in plasma glutamic acid.

At least in theory, the results might reflect an inhibitory effect of cell swelling on autophagic proteolysis resulting in a decrease in cellular amino acid release (Häussinger et al. 1993; Lang et al. 1998). Both histidine and arginine are broken down to glutamate, which is deaminated to produce urea when amino acid availability exceeds requirements for protein synthesis. The plasma urea concentration, which can be expected to decrease if proteolysis (Forman et al. 2012) and urea formation (Häussinger et al. 1993) decrease and renal urea clearance increases with water intake (Poortmans and Vanderstraeten 1994), tended to decline in the weeks of higher water intake, but did not reach statistical significance. It remains to be determined if water intake influences renal tubular amino acid transport, and/or amino acid accumulation in the renal medulla (Gullans et al. 1988; Hwang et al. 2010) modifies urinary amino acid excretion.

The particular sensitivity of plasma histidine, urine arginine, and urine glutamate excretion to change in hydration was unexpected. The authors are aware of histidine efflux in response to hypo-osmotic challenge in salmon and bacteria (Wong et al. 1995; Rhodes et al. 2010), but know of no other such data in humans.

Significant and consistent patterns of U-shaped change were not observed for other plasma or urine concentrations, including taurine, glycine, proline, serine, threonine, alanine, aspartate, and the sum of amino acids. Taurine efflux is observed after hypo-osmotic challenge in mammalian cells (Lang et al. 1998). Possibly, the urinary excretion and/or plasma concentration of other amino acids and osmolytes are affected only following marked, acute dehydration (Lang et al. 1998; Grant et al. 2000; Ordaz et al. 2004; Shennan et al. 2006). Depending on the cell type and timing of osmotic challenge, different amino acids may stand out (Horio et al. 1999).

## Limitations

Studies are needed to verify the present findings, resolve how they fit with the literature, and to determine their generalizability to other free-living conditions and population groups. The present study conditions, design, participants, background fitness levels and diet, and protocol were unlike conditions in previous in vitro studies or studies involving animals or patients. The participants in this study were healthy, free-living, young men, reporting less than 60 min/day moderate or vigorous exercise, and consuming a usual Western diet, including ready-to-eat foods, locally available for purchase (e.g., at supermarkets, food outlets). As the study diet was not controlled, dietary changes, such as the correlated decrease in other beverage intake, and/or intake of protocol-restricted foods such as caffeined beverages, may limit interpretation of the results. One participant (ID 5) admitted to caffeinated beverage intake midway through the study.

Further work is needed to pursue each of the four parameters identified as a potential biomarker of hyperosmotic stress on cells. The present analysis was not designed to characterize the biological mechanism(s) of any particular parameter. It does not account, for example, for specific amino acid availability in the diet, which is known to modify amino acid accumulation and efflux (Rhodes et al. 2010). This study design assumes that adaptive responses may require up to 14 days (Nielsen et al. 1993), and is informative only about 2-week consecutive periods. The protocol furthermore specified a 60–90 min delay between the water bolus and specimen collection. As some amino acids, such as taurine (Ghandforoush-Sattari et al. 2010), may be eliminated from plasma in a shorter interval of time, the 90 min delay in this study might have been too long to observe changes in some amino acid concentrations. Plasma histidine, the only plasma amino acid observed to change significantly and consistently across participants in this study, has a serum half-life over 90 min in healthy individuals (Sitton et al. 1988).

The study was motivated by reports of cell shrinkage-induced protein breakdown and cell swelling-induced amino acid efflux. Although the results were consistent with expectation that amino acid metabolism would change in response to change in chronic cell hydration, the results may not reflect change in efflux due to change in osmolyte accumulation. If the timing of specimen collection in this study missed amino acid efflux resulting from bolus-induced cell swelling, the observed changes might reflect change in other pathways. Change in plasma histidine, for example, might reflect change in ADH release.

For each parameter, the results suggested a possible cutoff for discriminating between chronic hypo- versus hyperosmotic stress. The data suggested a cutoff of 400 mmol/kg for the RBC deformability Ohyp, and a cutoff of 10 μmol/L for plasma histidine. For urine arginine and glutamic acid concentrations, possible cutoffs may be the assay detection limits. Further work is needed to confirm these cutoffs and determine their sensitivity and specificity. As decreases in these parameters are associated with disease states (Sitton et al. 1988; DeFranceschi et al. 1997; Watanabe et al. 2008), work is needed to define both lower and upper limits of the normal range for indices of chronic cell hydration.

## Summary

In summary, the present results warrant further work to determine if measures from blood or urine collected at one single time point can be used to index chronic cell hydration status. Consistent with expectation, the periods of chronically higher water intake were associated with repeated indication of less cell swelling, and selected lower plasma and urine amino acid concentrations in response to a hypo-osmotic challenge of 750 mL of drinking water. The changes associated with higher water intake are consistent with the study participants recovering from a baseline state of cellular adaptation to chronic hyperosmotic stress. Further work is needed to determine if and/or how RBC deformability, plasma or urine amino acid concentrations after a bolus of drinking water can be used to index chronic hyperosmotic stress on cells in epidemiologic studies.
